# Delayed Pseudoprogression in Glioblastoma Patients Treated With Tumor-Treating Fields

**DOI:** 10.7759/cureus.55147

**Published:** 2024-02-28

**Authors:** Norihiko Saito, Nozomi Hirai, Sho Sato, Morito Hayashi, Satoshi Iwabuchi

**Affiliations:** 1 Neurosurgery, Toho University Ohashi Medical Center, Tokyo, JPN

**Keywords:** electric fields, glioblastoma multiforme, temozolomide, radiation therapy, pseudoprogression, ttfields, glioblastoma

## Abstract

Tumor-treating fields (TTFields) is an established treatment modality for glioblastoma. False progression to chemoradiation is a known problem in patients with glioblastoma multiforme (GBM), with most cases occurring within three months of radiation therapy. In this report, we present two cases of delayed pseudoprogression caused by TTFields. Two patients with GBM who received TTFields showed signs of radiographic progression six months after the completion of radiation therapy. Patient 1 was a 37-year-old female with a glioblastoma in the right temporal lobe. Patient 2 was a 70-year-old male with glioblastoma in the left temporal lobe. Both patients received radiation therapy, followed by temozolomide (TMZ) maintenance therapy and TTFields. Patient 1 underwent a second resection; however, the pathology revealed only a treatment effect, and the final diagnosis was a pseudoprogression. In Case 2, the disease resolved with steroid therapy alone. In both patients, the lesions appeared later than during the typical pseudoprogression period. A recent study reported that TTFields increase the permeability of the plasma cell membrane, which may result in further leakage of gadolinium into the extracellular lumen. Further studies are needed to better characterize delayed pseudoprogression and improve treatment outcomes.

## Introduction

Glioblastoma, which accounts for approximately 9% of all primary intracranial tumors, has an extremely poor prognosis for brain tumors [1]. Although surgical removal of the tumor is as safe as possible and combination therapy with postoperative radiation therapy and temozolomide is the standard of care [2], glioblastoma remains a very intractable tumor, with many patients experiencing recurrence within one year and a median survival of less than two years [3-5]. Currently, research and development of novel therapies to overcome therapeutic resistance are still in progress. Among these, the usefulness of tumor-treating fields (TTFields) therapy, a novel therapeutic approach for glioblastoma, has been reported in many countries [6,7]. This therapy induces cell death by generating an alternating electric field through the application of a sheet containing electrodes to the scalp, which inhibits the formation of microtubules in glioblastoma cells, thereby arresting cell division.

Pseudoprogression is difficult to distinguish from tumor progression because the area of the enhanced effect expands early after radiotherapy with temozolomide (mainly within three months). Although imaging findings are suggestive of disease progression, most patients are asymptomatic, and the lesions often shrink thereafter [8]. In this report, we describe two cases in which patients treated with TTFields for glioblastoma developed pseudoprogression as late as six months after radiotherapy with temozolomide.

## Case presentation

Case one

**Figure 1:**
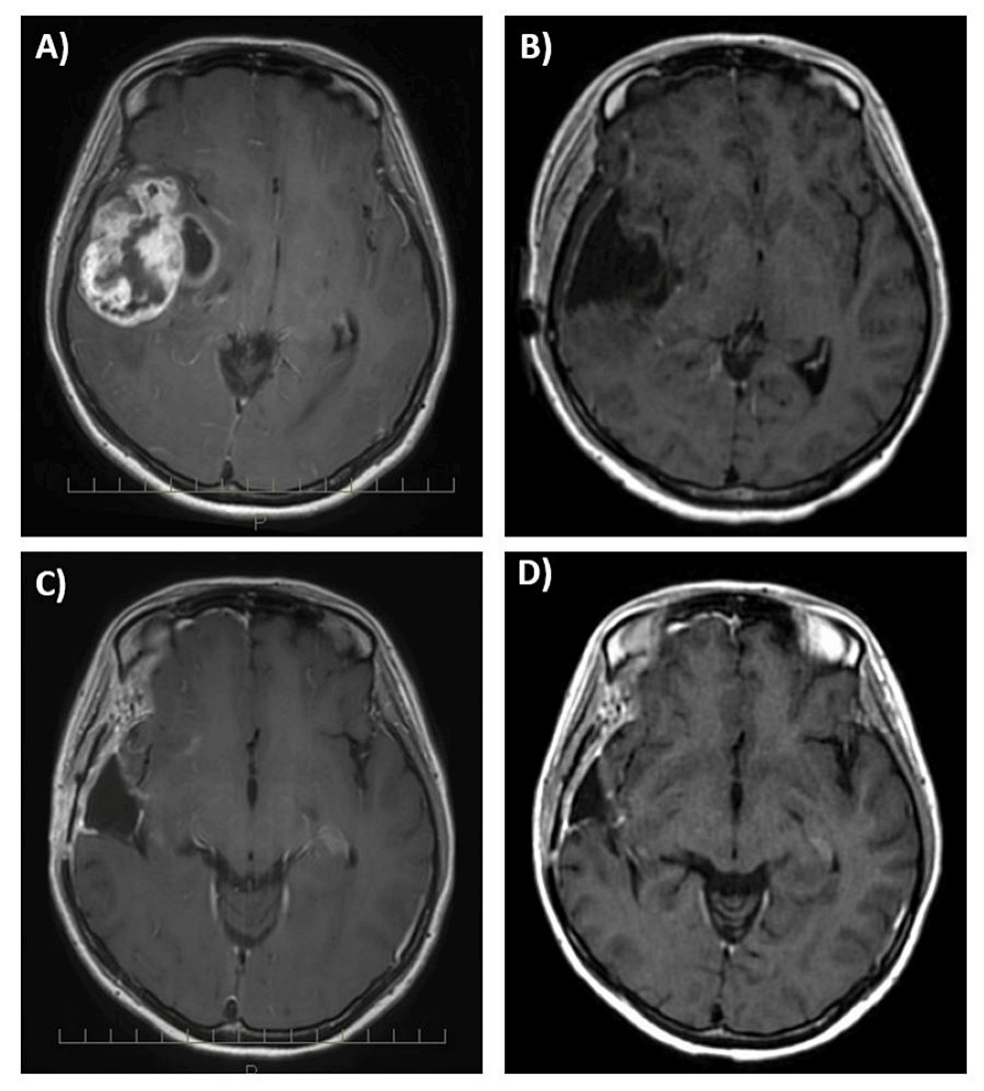
MRI brain findings (Case 1) (A) Preoperative MRI with contrast showing ring-shaped contrast-enhanced lesion was observed in the right temporal lobe; (B) Postoperative MRI with contrast after the tumor was surgically removed via craniotomy, showing total resection was achieved; (C) Two months after the start of maintenance therapy, no contrast lesions noted; (D) Four months after the start of maintenance, no contrast lesions noted.

A 35-year-old female presented to her former hospital with headaches. Magnetic resonance imaging (MRI) of the head revealed a brain tumor in the right temporal lobe, and the patient was referred to our hospital. A ring-shaped contrast-enhanced lesion was observed in the right temporal lobe (Figure 1A). The tumor was surgically removed via craniotomy, and total resection was achieved (Figure 1B). The pathological diagnosis was glioblastoma with wild-type IDH and unmethylated MGMT.

**Figure 2:**
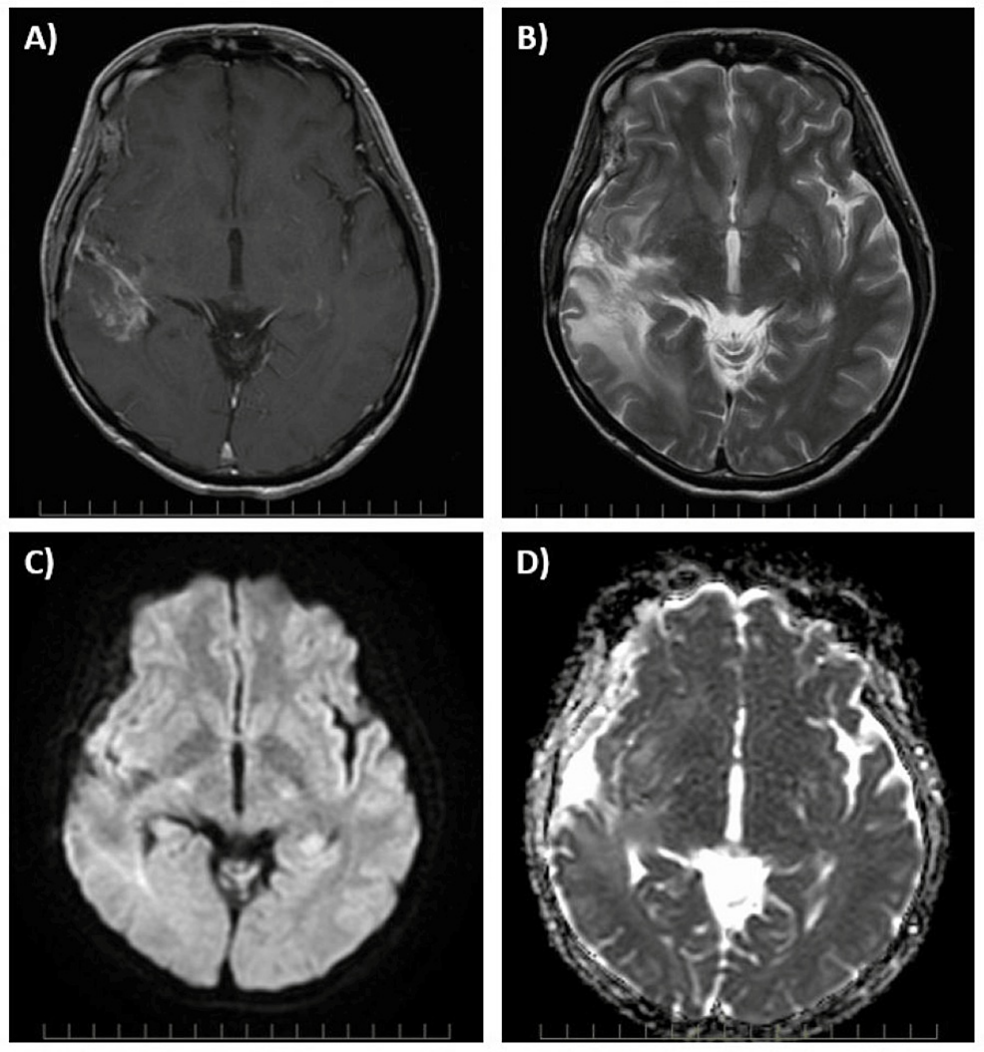
MRI brain findings at pseudoprogression six months following temozolomide plus TTFields therapy (Case 1) (A) The patient developed a new heterogeneously enhancing mass lesion in right temporal lobe on a contrast T1-weighted image; (B) T2-weighted image shows marked enlargement of brain edema; There was no diffusion restriction on diffusion-weighted image (C) or apparent diffusion coefficient map (D). TTFields: tumor-treating fields

**Figure 3:**
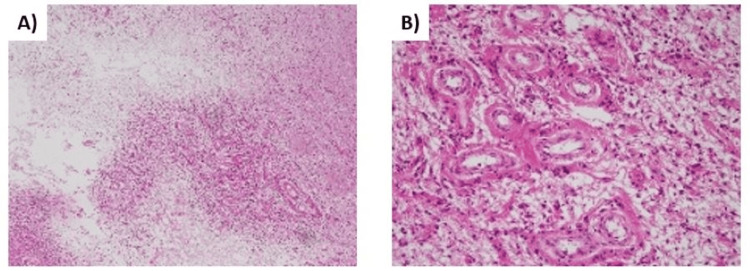
Histopathological findings (Case 1) (A) The area of necrosis appears hypocellular and sharply demarcated from the surrounding gliotic brain, H&E: 40x; (B) Histopathological examination of the lesion revealed degenerated brain tissue, hyalinized blood vessels (arrows), and coagulation necrosis with no evidence of tumor recurrence, H&E: 400x

The patient received standard treatment with temozolomide 75mg/m^2^ daily plus radiation therapy (60 Gy/30 fractions), followed by maintenance therapy with temozolomide 150 mg/m^2^ once a month for five days plus TTFields therapy. TTFields therapy was performed as follows: four transducer arrays were placed on the shaved patient's scalp and connected to a portable, battery- or power-operated device (NovoTTF™-100A; Novocure GmbH, Switzerland). Patients were trained on how to operate the device and then continued treatment at home. Treatment was continued while maintaining normal daily activities. The transducer array was changed once or twice a week by the patient or their caregiver. Routine MRI scans showed no tumor recurrence (Figure 1C, 1D). After a total of six cycles of temozolomide plus TTFields therapy, with respective mean compliance of 17.7 hours (73.8% of the day), MRI showed an increase in contrast-enhanced lesions without worsening neurological symptoms (Figure 2). Tumor recurrence was suspected, and reoperation was performed. Histopathological examination of the lesion revealed degenerated brain tissue, fibrotic microvessels, and coagulation necrosis with no evidence of tumor recurrence, and pseudoprogression was diagnosed (Figures 3A, 3B). The lesion resolved postoperatively and maintenance therapy without TTFields was continued. The patients had tumor recurrence 33 months following the initial diagnosis and died at 40 months. The patient did not receive TTFields therapy for tumor recurrence.　

Case two

**Figure 4:**
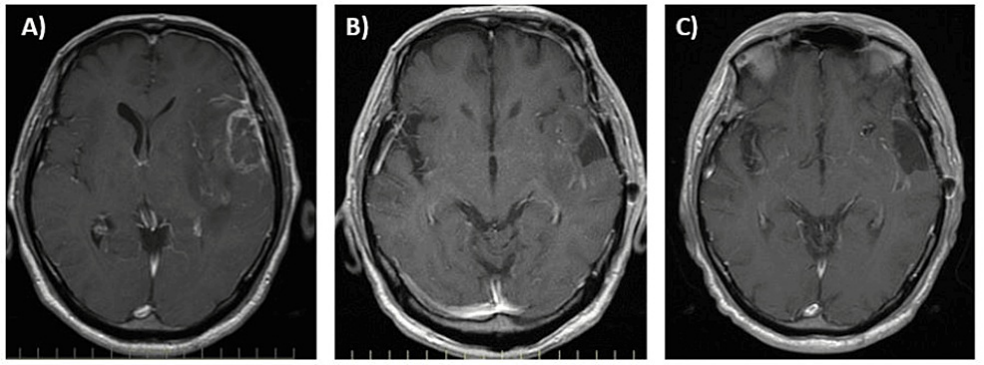
MRI brain findings (Case 2) (A) Preoperative MRI with contrast shows ring-shaped contrast-enhanced lesion in the left temporal lobe; (B) Two months after the start of maintenance therapy, no contrast lesions noted; (C) Four months after the start of maintenance, no contrast lesions noted.

A 70-year-old man presented at our hospital with aphasia. He was referred to our hospital because an MRI scan of his head revealed a brain tumor in the left temporal lobe. A ring-shaped contrast-enhanced lesion was observed in the left temporal lobe (Figure 4A). Craniotomy was performed to remove the tumor, and total resection was performed. The pathological diagnosis was glioblastoma with wild-type IDH and unmethylated MGMT. The patient received standard treatment with temozolomide 75mg/m^2^ daily plus radiation therapy (60 Gy/30 fractions), followed by maintenance therapy with temozolomide 150 mg/m^2^ once a month for five days plus TTFields therapy. The TTFields treatment was performed as in Case 1. Routine MRI scans showed no tumor recurrence (Figure 4B, 4C).

**Figure 5:**
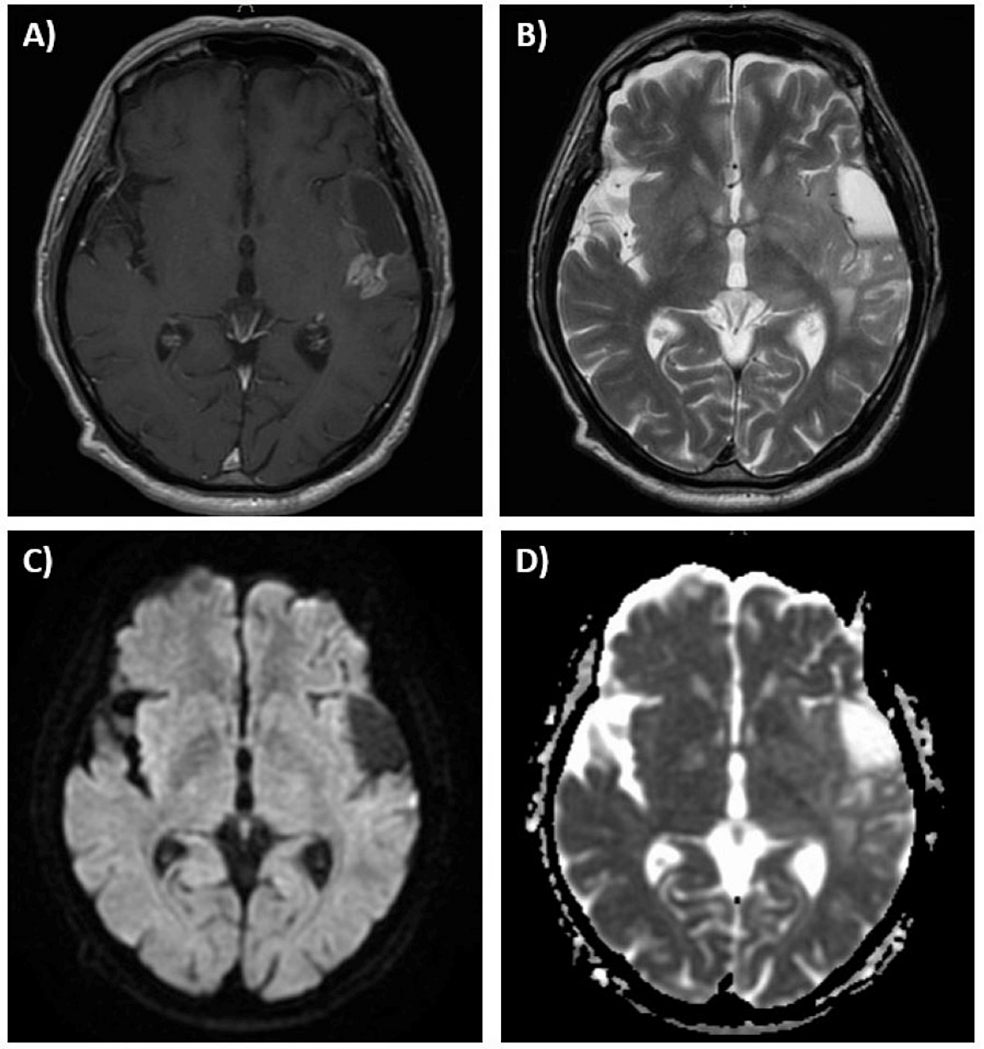
MRI brain findings at pseudoprogression six months following temozolomide plus TTFields therapy (Case 2) (A) The patient developed a new heterogeneously enhancing mass lesion in left temporal lobe on a contrast T1-weighted image; (B) T2-weighted image showed marked enlargement of brain edema; There was no diffusion restriction on diffusion-weighted image (C) or apparent diffusion coefficient map (D).

**Figure 6:**
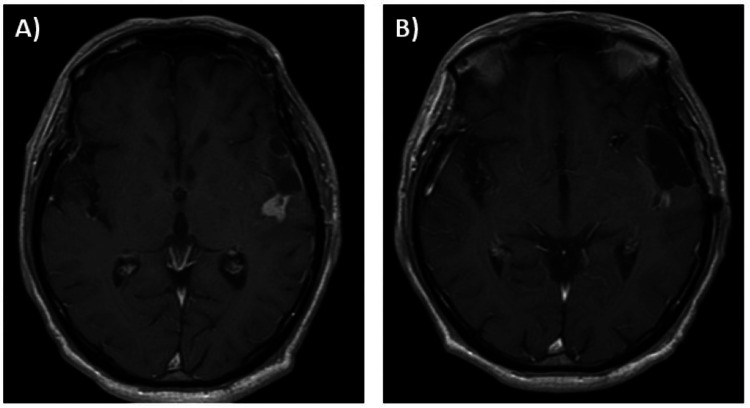
MRI brain findings (Case 2) (A) One months after steroid therapy, contrast lesion had shrunk; (B) Two months after steroid therapy, the contrast lesion had disappeared.

After a total of six cycles of temozolomide plus TTF therapy, with a respective mean compliance of 18.3 hours (76.2% of the day), the patient displayed no worsening of neurological symptoms; however, contrast-enhanced lesions were detected on MRI (Figure 5). Based on our experience with Case 1, we suspected pseudoprogression and administered dexamethasone 4 mg/day while continuing the temozolomide and TTFields therapy. Two months after administration, the contrast lesion had disappeared (Figure 6A, 6B). Then, temozolomide and TTFields therapy were administered for up to a total of 12 cycles and then completed. The patients had tumor recurrence 22 months following the initial diagnosis and died at 28 months. The patient did not receive TTFields therapy for tumor recurrence.

## Discussion

After radiation therapy with temozolomide, there was an increase in the contrast-enhanced area, especially within three months, which can be confused with tumor progression, a finding known as pseudoprogression. Pseudoprogression is associated with the use of temozolomide and occurs in approximately 6-30% of patients treated with chemoradiotherapy with temozolomide. The mechanism of pseudoprogression is thought to involve increased vascular permeability associated with tumor cells and endovascular cell damage, resulting in enhanced effects and cerebral edema.

Pseudoprogression occurs with a frequency of 20-30% after temozolomide combination therapy for glioblastoma, with the majority of cases occurring within three months of treatment, often without worsening neurologic symptoms [8]. Surgically confirmed necrosis without recurrent tumor was reported in seven of 51 (14%) patients with malignant glioma within six months of temozolomide chemoradiotherapy [9]. Regarding the timing of occurrence, a more similar study of 85 patients with malignant gliomas treated with temozolomide chemoradiotherapy reported pseudoprogression in 18 patients (21%), all on the first MRI performed within two months of treatment [10]. In addition, an analysis of 200 patients with IDH-mutated high-grade gliomas reported that 38 (19%) had pseudoprogression, occurring at a median of 10.5 months after radiation therapy [11].

The NovoTTF-100A system, a medical device that uses TTFields therapy, has been added to the treatment of glioblastoma. A low-intensity alternating electric field generated through a transducer array affixed to the head exerts a physical force on cellular components such as charged microtubules and centrioles, inhibiting microtubule formation and causing unbalanced mitotic and mitotic organelle deviation, thereby inhibiting the division of tumor cells and producing an antitumor effect.

In the clinical trial EF-14 for primary glioblastoma, NovoTTF in combination with temozolomide demonstrated a significant prolongation of both progression-free survival and overall survival in the NovoTTF treatment group compared with the temozolomide monotherapy group [7]. Based on the results of this research, TTFields therapy was approved in Japan, and the presented cases were treated as part of the reimbursement.

Two cases of pseudoprogression occurred six months after radiation therapy with temozolomide, which was later than usual. In the first case, the tumor was removed and histopathology showed no tumor recurrence; however, degenerated brain tissue, fibrotic microvessels, and coagulation necrosis were observed. In the second case, the lesion disappeared after steroid treatment, and a clinical diagnosis was made. The incidence of pseudoprogression has been reported to be higher in TTFields-treated patients than in untreated patients [12]. In our cases, overall survival in both cases was longer than the average for glioblastoma. Furthermore, recent studies have shown that TTFields increase the permeability of the cell membrane of tumor cells [13] and the blood-brain barrier in normal brain tissue [14,15], which may contribute to disease pathogenesis.

Continuous TTF therapy induces chronic inflammation, which further promotes this process mediated by transforming growth factor-β, tumor necrosis factor-α, and vascular endothelial growth factor-A, leading to the formation of abnormal microvessels [16,17]. As a result, we speculated that a delayed pseudoprogression may emerge in the form of microvascular insufficiency induced by TTFields therapy, accompanied by extensive and persistent damage to endothelial cells, ultimately leading to hypoxic tissue necrosis. Although the number of cases is small and it is difficult to determine the cause, we hypothesized that this observation may have been caused by the strong therapeutic effect of the combination of temozolomide and TTFields therapy. It is also possible that effects other than the antitumor effect of TTFields therapy, as has been the case in recent studies, may have contributed to the specific imaging findings. It is also possible that lesions that appeared to have recurred during TTFields therapy were pseudoprogressions, and pathologic diagnosis by minimally invasive techniques such as biopsy should be considered.

## Conclusions

Pseudoprogression is an abnormality described with temozolomide therapy that is difficult to distinguish from tumor progression because the area of enhanced effect expands at an early stage after radiation therapy with temozolomide. We reported two cases in which patients treated with TTFields for glioblastoma developed pseudoprogression as long as 6 months after radiation therapy with temozolomide.

Although imaging findings suggest disease progression, most patients are asymptomatic, so new contrast enhancement in patients receiving adjuvant temozolomide with TTFields should be judged with caution because of the possibility of pseudoprogression even later than the typical time. The pathologic diagnosis of pseudoprogression should be considered before changing therapy.
